# Does an Empty Nest Affect Elders’ Health? Empirical Evidence from China

**DOI:** 10.3390/ijerph14050463

**Published:** 2017-04-27

**Authors:** Min Gao, Yanyu Li, Shengfa Zhang, Linni Gu, Jinsui Zhang, Zhuojun Li, Weijun Zhang, Donghua Tian

**Affiliations:** 1 School of Social Development and Public Policy, China Institute of Health, Beijing Normal University, Beijing 100875, China; vivianhbs@126.com (M.G.); zhangshengfa1988@hotmail.com (S.Z.); gulinni@hotmail.com (L.G.); zhangjinsui1992@163.com (J.Z.); zhuojunlea@yeah.net (Z.L.); 2 School of Humanities and Social Sciences, North China Electric Power University, Baoding 071000, China; liyanyu2007@126.com

**Keywords:** empty nest, overall health, instrumental variable, limited-information maximum likelihood model (LIML), 2 stage least squares (2SLS)

## Abstract

The “empty-nest” elderly family has become increasingly prevalent among old people in China. This study aimed to explore the causality between empty nests and elders’ health using effective instrumental variables, including “whether old parents talk with their families when they are upset” and “ownership of housing”. The results showed that empty nests had a significantly adverse influence on elders’ physical health, cognitive ability and psychological health. Furthermore, urban elders’ cognitive ability was more influenced by empty nests than that of rural elders. Additionally, the effects of an empty nest on elders” health were more significant among female, single elders and senior rural elders. “Living resources”, “availability of medical treatment” and “social activity engagement” were found to be significant mediators between empty nests and elders’ health, accounting for 35% of the total effect.

## 1. Introduction

With the rapidly aging population in China, many older people are considered to have an “empty nest” [1]. The empty nest families mean that elderly do not live with their children or do not have a child. In fact, one-child policy in China was considered one of the major catalysts contributing to the large number of empty nest elders [2]. Since 2016, China cancelled its one-child policy and fully implemented the policy of allowing each couple to have two children as an active response to the aging population. Nevertheless, most of the new generation would not be able to have another child even if they wanted to because of their disadvantaged positions in the increasingly competitive job market and the increased costs of raising a child [3,4]. Some demographers have predicted that the ‘two-child’ policy will not lead to a baby boom [5,6,7].

In the traditional Chinese family pattern, when parents cannot take care of themselves, they can live with their children and receive assistance [8]. Recently, the “Data Analysis of the Sampling Survey of the Aged Population in Urban/Rural China 2010” report, published by China’s National Committee on Aging, indicated that the proportion of elderly people living alone was rising. More importantly, empty nest elders accounted for 49.3% of the entire older population, including 54.4% of urban empty nest elders and 45.6% of rural empty nest elders [9]. Compared with urban areas, the empty nest phenomenon has become increasingly serious in rural areas. Urbanization is now a global phenomenon and will inevitably affect the living patterns of the elderly in rural China. Based on the National New Type Urbanization Planning (2014–2020) report, there are approximately 100 million rural people who will move into cities from the country’s farming regions [10]. If this trend continues, the traditional family relations of being supported by children will certainly be challenged.

Whether there was a causal relationship between empty nest and Chinese elders’ health is an important issue that is well worth studying. Although there have been several studies on this topic, the findings have been equivocal and even conflicting, and the mechanisms between the two have remained unclear. Some studies have found that an empty nest has a protective effect on elders’ health [11,12] and that living alone provides a great opportunity for elders to enjoy their leisure time [13,14]. However, other studies have supported the hypothesis of a strong negative relationship between empty nest and elders’ health [15,16], especially regarding mortality risk [17] and changes in self-rated health [18]. Despite these divergent findings, there is strong evidence that empty nest is associated with elders’ health.

To more thoroughly assess the effects of empty nest on elderly health, physical health (ADL), cognitive ability (MMSE) and psychological health were adopted as indicators of health in this study. Some researchers have shown that empty nest elders have a better health condition than elders who live with their children [19,20,21]. In contrast, some studies have revealed that an empty nest has a significant adverse effect on elders’ health. A study from China showed that empty nest elders had worse self-care ability and lower mental health scores [22]. In addition, the prevalence of deterioration in physical function of the elderly has been associated with a low income, single status, poor living conditions, number of chronic diseases and poor family functional conditions. Some studies have indicated that an empty nest is negatively related with elders’ cognitive ability [23,24], especially for female, aged, rural, single elders and elders without health insurance [25]. In general, when children move out of their homes, old parents will be more frustrated, depressed and anxious. If these symptoms cannot be eliminated in time, they can weaken elders’ immunity and, more seriously, further increase their risk of cognitive disability and Alzheimer's disease [26]. The relation between empty nests and the psychological health of older people has attracted the attention of clinical researchers in recent years [27]. Some Chinese studies also found that empty nest elders had more discomfort, anxiety, and depression [21]. However, another study suggested that empty nest elders could enjoy more freedom when their children lived away from them and that they had more time to enjoy life, make friends and entertain [28].

Other studies have explored the indirect relationship between empty nests and elderly health [22,29]. Living resources, availability of medical treatment and social activities have been recognized as the three main potential mediating variables of this relationship. Empty nest elders were in worse condition because they did not have a sufficient pension (as farmers did not previously receive a pension in China) to support themselves; this situation led elders in impoverished mountainous regions in China to depend on their children [30]. When children live far away from their parents, they may not have the opportunity to provide sufficient material goods and assistance for their parents [31,32]. Living resources, a determining factor of health, have appeared to be significantly associated with an empty nest. Empty nest elders from low-income families have shown a tendency toward non-visiting and non-hospitalization after adjusting for need and predisposing factors [30,33]. Concurrently, previous studies have shown that younger age has strong associations with more social activities, which could prevent depression [34]. For the elderly, depressive symptoms can spontaneously appear when social support provided by work disappears and their children are unable to provide support for them in time [35].

Previous studies had some limitations. First, the endogenous problem was not adequately solved by the researchers who did explore the causality between an empty nest and elders’ health. Previous studies usually analyzed the effects of baseline living status on these elders’ health, after controlling for baseline health conditions [18,36], but ignored that the elderly could move in with their children if they felt unwell. Second, these studies have devoted more attention to the physical function of the elderly, although integrated studies on physical health, cognitive ability and psychological health have not been well conducted, especially in developing countries such as China [37]. Third, the effects of an empty nest on the health of the elderly may be heterogenous, which may have caused the deviation in prior understanding of the needs of elderly care services. For example, urban elders and rural elders have different ideas, life environments and family structures, and the relationship would certainly be biased if these two groups were combined [1,38]. Finally, almost no studies have examined the mechanism between empty nests and overall elderly health. Therefore, this study hypothesized that an empty nest had a negative effect on elders’ health in China, aimed to analyze the relationship between an empty nest and the overall health of the elderly, and explored the mechanisms behind how an empty nest influences the health of the elderly in urban and rural China by employing the method of instrumental variables, which was considered the best strategy to address the problem of endogeneity inherent to these types of data.

## 2. Materials and Methods

### 2.1. Data and Variables

#### 2.1.1. Data

Data from the Chinese Longitudinal Healthy Longevity Survey (CLHLS), which was conducted in 2008 and 2011, were used in this study. The CLHLS is the first national longitudinal survey of the oldest-old (aged 80 or over) in China, people aged 65–79 were included as a comparative group from 2002, and people aged 35–64 were further contained since 2008, covering approximately 85% of the total population in China [39,40]. The CLHLS sampling frame started with lists of all centenarians in the randomly selected counties/cities. For each centenarian, one octogenarian aged 80–89 living nearby, one nearby nonagenarian aged 90–99, and one nearby younger elder aged 65–79 of pre-designated age and sex were interviewed. The response rate was 97.7%. The data quality of the CLHLS was high, according to systematic assessments of the age reporting, reliability, validity, and consistency of numerous measures and the randomness of attrition [38]. Data are provided on respondents’ health conditions, daily functioning, self-perceptions of health status and quality of life, life satisfaction, mental attitude, and feelings about aging. In this study, subjects aged 65 years and over who were interviewed both in 2008 and 2011 were chosen, including 8418 subjects. Then, we deleted cases when independent variables, dependent variables, control variables or mediating variables in the sample were missing. The total missing cases were 595, accounting for 7% of the sample. Finally, a sample of 7823 subjects was included in this study, including 2931 urban elders and 4892 rural elders. The CLHLS study was approved by research ethics committees of Duke University and Peking University (IRB00001052-13074). All participants provided written informed consent. No experimental interventions were performed. An exempted Institutional Review Board (IRB) protocol was approved by Duke University (Pro00062871).

#### 2.1.2. Independent Variables

The presence of an empty nest was measured by the following question: who do you live with? Elders who lived alone or only with a spouse were defined as empty nest elders, which was coded as 1; those living with families (including children, grandchildren, sons-in-law, daughters-in-law, etc.) were defined as non-empty nest elders, which was coded as 0.

#### 2.1.3. Dependent Variables

Health was a multi-dimensional concept, including physical, cognitive and psychological health. Disability in activities of daily living (ADL) was defined as self-reported difficulty with any of the following activities: bathing, dressing, eating, indoor transferring, toileting, and continence. Each activity had three options: do not need help (coded as 3), need some help (coded as 2), and completely need help (coded as 1); the scores of six questions were summed (the total number between 6 and 18) and transformed it by the logarithm function to satisfy normality assumption [38,41,42]. In this study, Cronbach’s α based on standardized items was 0.886.

The Chinese version of the Mini-Mental Status Examination (MMSE) was used to measure cognitive ability [43] and included 25 easily understandable and answerable questions for the oldest-old Chinese population with normal cognitive functioning [40]. Moreover, it presented high reliability and validity in a previous study [44]. The total MMSE score was 25 (correct answers were coded as 1; incorrect answers were coded as 0). In order to avoid the errors caused by missing and unevenly distributed data, we added 1 to the MMSE score of everyone, then MMSE scores with logarithm were entered into the regression model as an interval variable owing to negatively skewed distribution of the original MMSE score. Other classifications of cognitive impairment were also tested and produced only minor differences in the results. In this study, Cronbach’s α of the MMSE based on standardized items was 0.884.

Psychological health (PH) was assessed by asking respondents seven questions, four of which were used to estimate the degree of elderly optimism, namely “I always look on the bright side of things,” “I like to keep my stuff clean,” “I can decide my life,” and “I am as happy as when I was younger.” The other questions were negative indicators and included sensitivity, “I often feel afraid and anxious”; loneliness, “I often feel lonely and isolated”; and self-rated capacity, “I feel more and more useless”. All responses ranged from “always” (coded as 1) to “never” (coded as 5). For the convenience of calculation, we reverse-coded negative feelings, and thus the higher the score was, the more positive the elder’s psychological health was rated on a 5-point Likert-type scale (“always” coded as 4, “never” coded as 0). The total range in psychological health score was from 0 to 28, we added 1 to the psychological health score of everyone in order to avoid missing values and unevenly distributed data, then transformed it by the logarithm function to satisfy normality assumption. In this study, Cronbach’s α based on standardized items was 0.8995.

#### 2.1.4. Control Variables

As shown in Table 1, to minimize confounding in the estimates of health, we adjusted for individual differences in personal characteristics, health awareness, community environment and area as proxy variables. Individual variables included age, gender, marital status, education, main source of income, income, medical insurance and recent health condition. Previous studies showed that our measures of education (i.e., any schooling) and income (i.e., annual income) were robust indicators of the socioeconomic resources available to older adults in China [38,44,45,46,47]. All variables were well validated and have been used frequently in the literature on empty nest elders’ health. Health awareness included smoking, drinking alcohol and exercising. Community variables reflected community services. Additionally, we classified China into eastern, central and western areas because different areas have different cultures and lifestyles. Bonferroni adjustments were carried out for each variable to account for multiple comparisons, Table 2 showed that there was a significant difference between empty nest elders and elders who lived with children.

#### 2.1.5. Mediating Variables

To determine whether a mediating effect between empty nests and elderly health existed, we selected living resources, availability of medical treatment, and social activities as potential mediators. Living resources was used to measure the abundance of money; we dichotomized this variable into “enough” (coded as 1) and “scarce” (coded as 0). To investigate the availability of medical treatment, the question “when you are ill, can you access timely medical treatment?” was asked, and the responses were dichotomized into “can” (coded as 1) and “cannot” (coded as 0). Similarly, the engagement of participation in social activities was represented by eight regular activities: housework, outdoor activities, having pets, reading books and newspapers, having livestock, playing cards, watching TV and listening to the radio, and other organized social activities. The responses ranged from “participate everyday (coded as 5)” to “never participate (coded as 1)”. Then, the scores of the eight questions were summed (range from 8 to 40), and higher scores indicated more active participation in social activities. Meanwhile, the scores were transformed by the logarithm function. In this study, Cronbach’s α based on standardized items was 0.642.

### 2.2. Model

Elderly living arrangement is associated with elderly health [48,49], as old people tend to move in with their children when they are in poor health [50,51,52]. However, when elders live with their families, families can provide daily care to improve elders’ health. Therefore, there may be endogeneity between the two, potentially introducing bias. Therefore, Fixed-effect Model (empty nest was considered an exogenous variable) and Limited-information Maximum Likelihood Model (LIML) were established, and Hausman’s test was adopted to examine the endogeneity based on the results of these two models. The results of Hausman’s test proved that an endogeneity problem existed (*p* < 0.05), which meant that an empty nest and elderly health influenced each other. Several studies showed that health condition was associated with the choice of living pattern. Healthy physical and mental condition could reduce the possibility of living with children [51,52,53]. Therefore, Instrumental variable (IV) methods were used to eliminate the bias introduced by the endogeneity.

Instrumental variables require a lack of association with residual items of the regression equation; additionally, they are supposed to be highly associated with the endogenous variable (empty nest) but not associated with the independent variable (elderly health). LIML models can avoid bias to some extent by selecting instrumental variables. Therefore, we chose LIML to estimate the association and to avoid an incorrect model, which may have led to parameter estimate bias [54]. However, LIML models will have a larger parameter estimation standard error because of potential multicollinearity problems, although larger samples and panel data can reduce the standard error of parameter estimation. Based on a large panel data, LIML models and instrument variables were employed to reduce the reverse effect of elders’ health on elders’ living pattern (live alone or not). Thus, the true effect of empty nest on elders’ health could be observed. In addition, the two-stage least squares method was used as a comparison to ensure the accuracy and credibility of the LIML results.

In this study, we selected “do you talk to your family when you are upset” (based on intergenerational spiritual support) and “ownership of housing” (the autonomy to choose one’s living pattern) as the potential instrumental variables, as they reflected mental support and material assistance. When elders are upset, families may be the most important source of spiritual support, and thus talking to families also indicated the quality of the relationship between the elders and their families [55]. If the relationship between elders and their families was not close, elders may have felt uncomfortable living with their children. Meanwhile, ownership of housing also had a great impact on elderly living arrangement [56,57]. In China, the tradition of living in one’s native land has been rooted in Chinese culture. If elders have a house of their own, they are more likely to live alone. Alternatively, if they do not, they may not feel a sense of belonging and may be more inclined to move in with their children. Therefore, the variable “ownership of housing” was related to where elders lived, rather than to their health. These two variables, “do you talk to your family when you are upset” and “ownership of housing”, were therefore selected as the instrumental variables.

Considering each health behavior separately, we assumed that there were two latent variables, which were defined as follows:

(1) $$Y_{it}=m\left(X_{it};\theta_{1}\right)+Z_{it}\theta_{2}+\mu_{i}+\varepsilon_{it}$$

(2) $$X_{it}=\beta_{1}Z_{i}+\beta_{2}C_{\mathrm{it}}+\omega_{i}+v_{it}$$

where $Y_{it}$ represents the three indicators of elder’s health for the individual *i* in time period *t*, and $X_{it}$ is the empty nest variable. $Z_{i}$ includes covariant variables representing individual characteristics. $C_{\mathrm{it}}$ are the instrumental variables. $v_{i}$ represents residuals. m denotes a polynomial of known length with unknown coefficients $\theta_{1}$, while $\beta^{\prime }$ ($\beta_{1}$, $\beta_{2}$) and $\theta^{\prime }\left(\theta_{1},\;\theta_{2}\right)$ are parameters to be estimated.

We first estimated the raw correlation between elder’s health ($Y_{it}$) and the empty nest variable ($X_{it}$) for a man i at time t as in (1). The IV method was employed to address the endogeneity between the health variable and the empty nest variable, which affected the variation in the empty nest variable generated by the variables “talk to families” and “ownership of housing”.

During analysis procedure, regression results of two stages of LIML model and 2SLS model were reported. Results of the first stage showed that the effects of instrumental variables on variable “empty nest”; Results of the second stage showed that the effects of variable “empty nest” on elderly health after adopting instrumental variables. Besides, according to the empirical rule proposed by Staiger and Stock, in IV regression, if the F values of the first stage are greater than 10, the weak instrumental variable problem can be excluded [58].

In addition, the weak instrumental variable problem was estimated based on ${\mathrm{Cov}\;}_{\left(X_{\mathrm{it}},\;C_{\mathrm{it}}\right)}$ and was improved when ${\mathrm{Cov}\;}_{\left(X_{\mathrm{it}},\;C_{\mathrm{it}}\right)}\approx 0$. Therefore, LIML, which was not sensitive to weak instrumental variables when estimating the relationship between an empty nest and elderly health, was further chosen to solve the problem of a weak instrumental variable. In large samples, the results of LIML models and 2SLS models were similar. However, the former model had fewer errors than the latter model when weak instrumental variables existed. Meanwhile, the over-identification test was used to verify the effectiveness of the instrumental variables because the number of instrumental variables was greater than the number of internal variables. If the detection *p*-value > 0.05, it meant that excessive recognition didn’t exist. We proposed another hypothesis, which stated that living resources, availability of medical treatment and social activities may mediate the relationship between an empty nest and elders’ health based on literature review. The traditional Causal Step Approach was adopted to examine and calculate the mediating effect, and a mediating effect model was constructed:

(3) $$Y_{it}=c*X_{it}+Z_{it}\theta_{2}+e_{1}$$

(4) $$M_{it}=a*X_{it}+Z_{it}\theta_{2}+e_{2}$$

(5) $$Y_{it}=c^{\prime }+b*M_{it}+Z_{it}\theta_{2}+e_{3}$$

In the equations, $Y_{it}$ represents elders’ health, $X_{it}$ represents the empty nest, $M_{it}$ represents the mediator variables (living resources, availability of medical treatment and social activities). c represents the total effect of an empty nest on elders’ health. a represents the effect of empty nest on mediator variables ($M_{it}$). b represents the effect of mediator variables ($M_{it}$) on elders’ health. $c^{\prime }$ represents the effect of empty nest on elders’ health after controlling for the mediator variables. The calculation of mediating effect is as follows:

(6) E=ab/c

If $a,\;b,\;c,\;and\;c^{\prime }$ pass the significance test, it means that mediating effect exists. If $c\;and\;c^{\prime }$ both do not pass, it means that the estimated parameters have no statistical significance. If either a or b does not pass, Sobel’s test is required:

(7) $$Z=\hat{a}\hat{b}/S_{ab}$$

(8) $$S_{ab}=\sqrt{{\hat{a}}^{2}S_{b}^{2}+{\hat{b}}^{2}S_{a}^{2}}$$

$\hat{a}$ and $\hat{b}$ represent the estimation of a and b. $S_{a}$ and $S_{b}$ represent the standard error of $\hat{a}$ and $\hat{b}$ based on the Z value of $S_{ab}$ and a review of the standard normal distribution table. If the value of *p* is less than 0.1, it means that there is a mediating effect [59].

## 3. Results

A significant difference between empty nest elders and non-empty nest elders was found in the primary data analysis. The results of Hausman’s test proved that endogeneity existed (*p* < 0.05); therefore, the LIML model was used to address this problem. Concurrently, the relationship and mechanism between an empty nest and elder’ health were also investigated in an urban sample and a rural sample.

### 3.1. Sample Characteristics

Analysis of variance was employed to compare the differences in ADL, MMSE and psychological health between empty nest elders and non-empty nest elders, and the sample characteristics are shown in Table 2. Of the total sample, elders living with children accounted for most of the respondents. In 2008 and 2011, empty nest elders’ ADL (M_2008_ = 2.882, M_2011_ = 2.846), MMSE (M_2008_ = 2.938, M_2011_ = 2.924) and PH (M_2008_ = 2.876, M_2011_ = 2.888) scores’ logarithm were significantly higher than those of non-empty nest elders. Moreover, non-empty nest elders were about six years older than the empty nest elders (M_2008_ = 79.594, M_2011_ = 81.569), which may be another reason why the empty nest elders were healthier.

Compared with the group of non-empty nest elders, empty nest elders had significantly more years of education (M_2008_ = 2.689, M_2011_ = 2.886), were more often their own main source of income (M_2008_ = 0.250, M_2011_ = 0.281), more often had a spouse (M_2008_ = 0.624, M_2011_ = 0.589), and smoked (M_2008_ = 0.235, M_2011_ = 0.214) and exercised (M_2008_ = 0.367, M_2011_ = 0.405) more often. However, non-empty nest elders had a significantly higher income (M_2008_ = 8.696, M_2011_ = 8.724).

Based on the results of previous studies, the variables, including “living resources”, “availability of medical treatment” and “social activities”, were used as potential moderators of the relationship between empty nests and elders’ health. Specifically, a lack of living resources can definitely damage elders’ health [25]. If the elders could not obtain medical treatment when they were sick, this would also directly influence their health. Additionally, empty nest elders’ ability to engage in social activities may be related to their psychological health. A previous study showed that the psychological health of empty nest elders cannot be guaranteed and that their communications with friends could also be reduced because of a lack of support from social networks [60]. The results of the statistical analysis showed that inadequate living resources (M_2008_ = 0.759, M_2011_ = 0.772) and underutilization of medical treatment (M_2008_ = 0.955, M_2011_ = 0.909) were significantly more prevalent in empty nest than in non-empty nest elders. However, empty nest elders engaged in more social activities (M_2008_ = 2.949, M_2011_ = 2.924) in this sample. Empty nests may have an indirect influence on elders’ health through these three moderators.

### 3.2. Empty Nest Elders’ ADL

Table 3, Table 4 and Table 5 present the findings of the LIML analyses compared with the 2SLS analyses. In this study, the F values in the weak identification test of the LIML model were 13.547 for the urban group and 46.875 for the rural group, which means there was no need to worry about weak instruments. Additionally, when the number of instrumental variables is more than that of endogenous variables, over-identifying restriction tests should be required. In this study, the *p* values of over-identifying restriction were 0.863 for the urban group and 0.138 for the rural group, meaning there was no problem with over-identification. After resolving the endogeneity problem, the results of the second stage of the LIML model showed that urban empty nest elders had significantly poorer health compared with non-empty nest elders (β = −0.617, *p* < 0.001). Furthermore, similar results were obtained in the 2SLS analyses (β = −0.616, *p* < 0.001), which further verified the robustness of the regression. When elders lived with their children, they could receive more daily care from their children; however, empty nest elders had to rely on themselves, and this placed a substantial amount of pressure on their health. In the rural sample, an empty nest also had a negative influence on elders’ ADL (β = −0.238, *p* < 0.001). In addition, education years (β = −0.002, *p* < 0.01) and exercising (β = 0.051, *p* < 0.001) were also significantly associated with rural elders’ ADL.

In the urban sample, elders’ ADL scores seemed to decline gradually as age progressed (β = −0.008, *p* < 0.01), that is, young elders had a better health condition. However, income (β = −0.050, *p* < 0.001) and being ill in the past two weeks (β = −0.081, *p* < 0.001) were negatively related to elders’ health, whereas having a spouse (β = 0.159, *p* < 0.001) and exercising (β = 0.089, *p* < 0.001) were positively related to elders’ health.

### 3.3. Empty Nest Elders’ Cognitive Ability

The effects of an empty nest on urban and rural elders’ MMSE scores are shown in Table 4; as expected, an empty nest had a negative relation with cognitive ability in urban elders (β = −3.585, *p* < 0.001) and rural elders (β = −2.438, *p* < 0.001). Furthermore, in the urban sample, elders who were male (β = 0.224, *p* < 0.01), younger (β = −0.045, *p* < 0.01) and those whose major source of income was mainly themselves and their spouses (β = 0.260, *p* < 0.01) had significantly higher cognitive ability. Meanwhile, spousal support (β = 0.931, *p* < 0.001) and exercising (β = 0.326, *p* < 0.001) also influenced cognitive ability significantly. However, elders who had been ill in the past two weeks had significantly poorer cognitive ability (β = −0.206, *p* < 0.01). In the rural sample, an empty nest had a dramatic positive impact on elders who were male (β = 0.291, *p* < 0.001), younger (β = −0.046, *p* < 0.001), married (β = 0.540, *p* < 0.001), whose main source of income was themselves (β = 0.226, *p* < 0.001) and who had a low income (β = −0.195, *p* < 0.001). Besides, elders who had not been ill in the past two weeks (β = −0.124, *p* < 0.001) and exercising (β = 0.204, *p* < 0.001) had higher cognitive ability.

### 3.4. Empty Nest Elders’ Psychological Health (PH)

Psychological health is another important part of elders’ health, and the effects of an empty nest on urban and rural elders’ psychological health are shown in Table 5. The results of the LIML regression indicated that an empty nest had a significant influence on urban elders’ PH (β = −3.751, *p* < 0.001) and rural elders’ PH (β = −2.595, *p* < 0.001). The results of the 2SLS regression further demonstrated that an empty nest had a significantly negative influence on elders’ PH. Meanwhile, we also found that an empty nest had a greater effect on urban elders’ PH than on rural elders’ PH.

In the urban sample, elders who were older (β = −0.037, *p* < 0.001), single (β = 0.979, *p* < 0.001), sick in the past two weeks (β = −0.238, *p* < 0.001) and had a higher income (β = −0.287, *p* < 0.001) had significantly poorer PH. However, exercising (β = 0.309, *p* < 0.001) and having a main source of income of themselves and their spouses (β = 0.303, *p* < 0.01) improved the PH of elders. Similar results were also found in the rural sample.

### 3.5. Heterogeneity Analysis

The previous results showed that an empty nest had an effect on elderly health. From the results of regressions, elders with different gender, age, marital status and exercise status had significant differences in health. Hence, interaction analysis was performed to estimate the association between two factors. In Table 6, significant differences between rural and urban elders were not observed with respect to gender (*p* > 0.05) and age (*p* > 0.05), but they were noted among marital status (*p* < 0.001) and exercise status (*p* < 0.001). The proportion of rural elders who were single (*p* < 0.001) or did not exercise (*p* < 0.001) was significantly higher than that of urban elders. In terms of gender, the proportion of women who were over 75 years of age (*p* < 0.001), single (*p* < 0.001), or did not exercise (*p* < 0.001) was significantly higher than that of male elders. For senior elders, the proportion of elders who were single (*p* < 0.001) or did not exercise (*p* < 0.001) was significantly higher. For single elders, the proportion of elders who did not exercise was significantly higher (*p* < 0.001).

Table 6 showed that characteristics of the individual were different among different subgroups, it might lead to the effects of an empty nest on subgroups were different. Therefore, the effect of an empty nest on elders’ health among different subgroups was further analyzed.

#### 3.5.1. Activities of Daily Living (ADL)

Table 7 showed that in the urban sample, in terms of gender, female empty nest elders had significantly lower ADL scores (β = −0.472, *p* < 0.01), but for male elders, regardless of whether they lived with their children, their ADL scores were not related to the empty nest variable (*p* > 0.1). For age, an empty nest had a weak association with 65–74 years of age elders’ ADL (*p* > 0.1), but had a strong association with 65–74 years of age elders’ ADL (β = −0.681, *p* < 0.01). In terms of marriage, an empty nest was not associated with elders’ ADL when they lived with a spouse (*p* > 0.1), but for single (including unmarried, divorced or widowed) elders, an empty nest had a negative impact on their ADL (β = −0.448, *p* < 0.001). In addition, urban empty nest elders who did not exercise had poor physical health (β = −1.031, *p* < 0.01), whereas for elders who did exercise, an empty nest was not related to elders’ ADL. In the rural sample, gender, age, marital status and exercising still had a significant influence on elders’ ADL. Elders who were female (β = −0.237, *p* < 0.001), were aged more than 75 years (β = −0.287, *p* < 0.001), were single (β = −0.200, *p* < 0.001) or did not exercise (β = −0.320, *p* < 0.001) were more negatively affected by an empty nest.

#### 3.5.2. Mini-Mental State Examination (MMSE)

Table 7 showed that in the urban sample, an empty nest could impair the cognitive ability of elders who were female (β = −2.727, *p* < 0.001) and 65–74 years of age (β = −3.966, *p* < 0.001). However, in terms of marriage, an empty nest was weakly associated with the MMSE scores of married elders (*p* > 0.05), whereas it had a significantly negative effect on the MMSE scores of single elders (β = −2.623, *p* < 0.001). For elders who did not exercise, an empty nest had a strong relationship with their MMSE scores (β = −6.175, *p* < 0.01).

In the rural sample, an empty nest had a significantly negative influence on elders, male (β = −2.672, *p* < 0.001) and female (β = −2.355, *p* < 0.001). Meanwhile, an empty nest had also a significant negative influence on the MMSE scores of elders who were over 75 years of age (β = −2.906, *p* < 0.001) or single (β = −2.171, *p* < 0.001). Moreover, regardless of whether elders exercised (β = −1.550, *p* < 0.01) or not (β = −2.738, *p* <0.001), an empty nest impaired their cognitive ability.

#### 3.5.3. Psychological Health (PH)

In Table 7, Empty nests had a strong adverse impact on elders’ psychological health, especially for female (β = −3.008, *p* < 0.001) over 75 years of age (β = −4.315, *p* < 0.001) and single (β = −2.749, *p* < 0.001) urban elders; however, for married urban elders, the influence of an empty nest was positive (β = 2.315, *p* < 0.01). For elders who did not exercise, an empty nest had s significant negative influence on elders’ psychological health (β = −6.664, *p* < 0.01).

In the rural sample, over 75 years of age (β = −3.121, *p* < 0.001), or single (β = −2.311, *p* < 0.001) showed negative associations with an empty nest. The effect of empty nest on elders’ psychological health was no significant difference between male (β = −2.807, *p* < 0.01) and female (β = −2.538, *p* < 0.001). Moreover, Elders who did not exercise (β = −2.967, *p* < 0.001) were more negatively affected by an empty nest.

### 3.6. Mediating Effect Analysis

Based on the results above, an empty nest had a direct negative effect on elders’ health. One thing worth noting is that an empty nest is a choice of living arrangement, and many factors may be significantly associated with it. Therefore, we hypothesized that there may be a mediating effect between an empty nest and elders’ health. Based on previous studies, this study aimed to explore mediating variables from the material well-being, health services and social activities perspectives. First, material well-being assistance was considered, as if the elders lived alone, they may receive less material assistance from their children. Furthermore, elderly people are accustomed to saving money, and they may only buy basic living goods to keep from starving. However, if elders did not have enough money or basic living goods to remain healthy, then an empty nest could directly impair their health. Second, the utilization of medical services plays a crucial role in keeping old people healthy. If elderly people lived alone, their accessibility and utilization of medical services could be reduced. For example, it may be inconvenient for them to go to hospitals without being accompanied by their children, and the distance to the hospital and the complicated medical procedures may present a challenge and barrier to receiving medical care. Third, social activities were included because for elderly people, participating in social activities not only improves their health, but more importantly, they can receive spiritual support by making friends with other people, further promoting a good state of mind. If they live alone, they may have to perform more housework and thus have less time to participate in social activities.

As shown in Table 8, an empty nest not only had a direct influence on elders’ health but also had an indirect influence on elders’ health. For urban elders, “living resources” (Z = −2.715, *p* < 0.01) and “medical treatment” (Z = −2.527, *p* < 0.01) had significant mediation effects between an empty nest and elders’ ADL, accounting for 10% of the total effect. For rural empty nesters, “living resources” (Z = −3.211, *p* < 0.001), “medical treatment” (Z = −2.082, *p* < 0.05) and “social activities” (Z = −3.371, *p* < 0.001) also had a strong mediating effect, accounting for 24% of the total effect. In addition, “social activities” played the most important role, accounting for 15.87%.

Furthermore, these three mediator variables also had significant mediation effects on the relationship between an empty nest and the MMSE and PH scores of elderly people, although the degrees of the effect declined. In urban and rural elders, “living resources” (in rural, 3.61%; in urban, 4.36%) and “medical treatment” (in rural, 4.12%; in urban, 4.84%) acted as significant mediators. In the rural sample, the “social activities” variable exerted a main mediating effect (Z = −3.381, *p* < 0.001, M = 9.08%). In terms of elders’ PH, “social activities” showed a main mediating effect between an empty nest and rural elders’ PH (Z = −3.361, *p* < 0.001, M = 5.79%). In contrast, in urban elders, “medical treatment” contributed to the main mediating effect (Z = −3.616, *p* < 0.001, M = 8.33%).

## 4. Discussion

In this study, sample characteristics showed that empty nest elders’ ADL, MMSE and PH scores’ logarithm were significantly higher than those of non-empty nest elders. These results may be influenced by selection bias, as healthier elders were capable of taking care of themselves and living independently may have been their best choice for enjoying the leisure of retirement, whereas elders with poor health had to accept assistance from their families. After selecting “whether old parents talk with their family when they are upset” and “ownership of housing” as effective instrumental variables to address the endogeneity problem, we explored the causality between an empty nest and elderly health. To the best of our knowledge, this may be the first study to explore whether an empty nest influences the overall health of Chinese urban and rural elders, and this study also identified the mechanisms between an empty nest and elderly health.

It was found that an empty nest had a significant negative influence on urban (β = −0.617, *p* < 0.001) and rural (β = −0.238, *p* < 0.001) elders’ physical health, especially for disadvantaged elders (female, older, single). Moreover, urban (β = −3.585, *p* < 0.001) and rural elders’ (β = −2.438, *p* < 0.001) cognitive ability was affected by an empty nest. Exercising could offset the negative influence of an empty nest on cognitive health. Furthermore, the results revealed that an empty nest had a significantly negative influence on urban elders’ PH (β = −3.751, *p* < 0.001) and rural elders’ PH (β = −2.595, *p* < 0.001).

Previous studies showed that living arrangements were relevant to elderly health [19,20,61], and some studies demonstrated that elderly health was a key factor influencing elderly living arrangement [36,62,63]. In this study, solving the endogeneity problem was essential. However, the interaction between elderly health and living arrangement has still not received enough attention in previous studies. To assess the true effects of an empty nest on elderly health, the instrumental variable method, which is an appropriate solution to addressing the endogeneity problem, was used in this study. A recent study used “the first child of the old person is a son and has survived” as an instrumental variable to resolve the endogeneity between an empty nest and elderly health, but along with changes in procreation and conception and the decreasing number of newborns, this instrumental variable does not have long-term applicability [25]. “Talks to family” and “ownership of housing” have more extensive applicability.

This study examined the causality between empty nests and elders’ physical health, cognitive ability and psychological health using a large-scale, nationwide longitudinal survey of elders in China in 2008 and 2011. A key finding of this study was that an empty nest had a significantly negative influence on urban and rural elders’ ADL, MMSE and PH scores. Elders who lived with their children could receive daily care, economic support and spiritual consolation from their children and therefore had better health conditions [16,23,24,64]. However, empty nest elders received less help from their families and society, especially in the case of certain elders, such as female, older, single elders and who may receive less help and thus have relatively worse health conditions.

Regarding elders’ cognitive ability, when elders lived with children, they received daily life assistance from their children, and their communication with their family may have also helped mitigate the deterioration in cognitive ability. However, when elders lived alone, they spent more time and energy taking care of themselves, and their cognitive ability received less attention. Another important finding was that exercising could offset the negative influence of an empty nest on health. Exercising was also a type of social interaction, as during exercise, elders could communicate with each other and continually accept new things, which may help in reducing deterioration in cognitive ability [65,66]. Furthermore, the findings also revealed that an empty nest had a significantly negative influence on elders’ psychological health, especially that of urban elders. A possible reason for this finding may be that when elders began to live alone, they might feel abandoned by their children. It could produce emotional problems, including discomfort, anxiety, pain, and even depression [21,67].

The effects of an empty nest on elders’ health differed between male and female elders. For females, elders who were older (aged 75 years and over), single and did not exercise were more significantly influenced by an empty nest, and this may be because they were in a disadvantaged position regarding their health. For elderly people with an older age, it is clear that they are relatively vulnerable to illness due to a deterioration in physiological function [68]. In addition, the old rural elderly would be more harmed by an empty nest. For rural elders, farming had been an important part of their daily lives, and living with children would directly affect old men’s workloads, further influencing their health. In China, females usually provide daily care services, and when elderly people live alone, they may receive less assistance from their families [69,70]. From the perspective of family structure, empty nest couples have to depend on each other, and thus spouses play a very important role in providing daily care and spiritual support [71,72]. Compared with married empty nest elders, single empty nest elders may live alone because they had no family members around to provide emotional support [73]. Although China has made great economic advances, its social security system for elderly people remains under-developed [74]. To prevent tragic outcomes in empty nest elderly people, the screening and treatment of depressive symptom should be strengthened; additionally, the responsibility of the family should be intensified because the family still provides an important role in supporting elders, especially because a specific elderly care system had not been established in China [75].

There was a difference in the effects of an empty nest between urban elders and rural elders. Compared with rural elders, urban elders’ health was more influenced by an empty nest. In rural China, the long-term farming lifestyle, although the elders are out of the labor market, have made them used to farming work such as planting crops, which not only provides food but also keeps them healthy. In addition, rural people may have closer neighborhood relationships than urban people, which may help alleviate the psychological pressure of rural elders [76].

Another finding was that an empty nest also had an indirect influence on empty nest elders’ health through living resources, availability of medical treatment and social activities. The exploration of potential mediating effects was another important contribution of this study. To date, there have been no studies investigating the effect of the mediator variables “living resources”, “availability of medical treatment” and “social activities” on the relationship between an empty nest and elders’ health. The results showed that all three variables played important mediating roles in the relationship between empty nest and elders’ health. Results of Sobel test reported that in urban elders, the effects of the mediator variables “living resources” and “availability of medical treatment” were greater on the relationship between an empty nest and elders’ health; while in rural elders, the effect of mediator variable “social activities” was more important.

This study provided empirical evidence regarding empty nest elders: empty nest elders should not only receive attention in terms of their material needs but also regarding their participation in more social activities for relaxation and entertainment. In the long term, an elderly care system should be established as soon as possible in China to take care of the increasing number of elderly people, given the lower birth rates and the longer life expectancies in the future. It is clearly impossible to sustainably enhance the quality of life of elderly people by relying only on family members. This study also emphasized the importance of developing a social security system for aged people that not only provides economic support and medical aid for empty nest elders but also pays attention to empty nest elders’ spiritual needs and social activities to allow elderly people to maintain a good health status.

There are some limitations of this study that should be acknowledged. First, the data were based on the best available data on the population; the analysis used convenient data rather than data specifically designed for empty nest elders, and thus related details may not be included. Second, mediator variables preclude subjectivity and variability. In this study, only three variables were chosen as mediator variables based on the data and our knowledge, and there may be other potential variables that were neglected.

## 5. Conclusions

In conclusion, empty nests had a significantly adverse influence on elders’ health, especially for the following respondents: female elders, elders over 75 years old age, single elders and elders did not exercise. The findings from this study explored the pathways underlying these effects in old people. “Living resources”, and “availability of medical treatment” acted as significant mediators between an empty nest and elders’ health, and “social activity engagement” exerted a main mediating effect in the rural sample. The findings contribute to a growing body of literature exploring whether empty nests are a risk factor for elderly health.

## Figures and Tables

**Table 1:** Control variables.

Age	65–114
Gender	(1 “male”; 0 “female”)
Marital status	(1 “married”; 0 “single (including unmarried, divorced or widowed)”)
Education years	0–24
Main source of income	(1 “myself and spouse”; 0 “others”)
Income	(ln (Annual Household Income))
Medical insurance	(1 “have”; 0 “do not have”)
Sickness in the past two weeks	(1 “yes”; 0 “no”)
Smoking	(1 “yes”; 0 “no”)
Drinking alcohol	(1 “yes”; 0 “no”)
Exercising	(1 “yes”; 0 “no”)
Having community services	(1 “yes”; 0 “no”)
Area	(1 “east”; 2 “central”; 3 “west”)

Note: The distribution of “Age” was relatively normal, so we did not process “Age” with logarithm; many elders are illiterate, and if we had processed “Education years” with logarithm, many data points would be deleted.

**Table 2:** Sample description and analysis of variance, CLHLS 2008–2011.

	2008				2011			
Dependent Variables	Empty Nest Elders		Non-Empty Nest Elders		Empty Nest Elders		Non-Empty Nest Elders	
	Mean	SD	Mean	SD	Mean	SD	Mean	SD
ADL	2.882 *	0.057	2.857	0.117	2.846 *	0.148	2.771	0.238
MMSE	2.938 *	0.553	2.692	0.887	2.924 *	0.647	2.554	0.997
PH	2.876 *	0.558	2.682	0.846	2.888 *	0.567	2.640	0.914
**Control variables**								
Gender	0.543 *	0.498	0.388	0.487	0.553 *	0.497	0.393	0.488
Age	79.594 *	9.264	84.886	11.295	81.569 *	9.238	88.147	10.900
Marital status	0.624 *	0.484	0.299	0.458	0.589 *	0.492	0.264	0.441
Education years	2.689 *	3.674	2.068	3.398	2.886 *	3.771	1.990	3.324
Main source of income	0.250 *	0.433	0.187	0.390	0.281 *	0.450	0.195	0.396
Income	8.696 *	1.613	9.576	1.139	8.724 *	2.463	9.784	1.794
Medical insurance	0.074	0.262	0.066	0.248	0.856	0.351	0.835	0.371
Sickness in the past two weeks	0.166	0.372	0.145	0.352	0.208	0.406	0.225	0.418
Smoking	0.235 *	0.424	0.181	0.385	0.214 *	0.410	0.164	0.370
Drinking alcohol	0.220	0.414	0.181	0.385	0.193	0.395	0.159	0.366
Exercising	0.367 *	0.482	0.305	0.461	0.405 *	0.491	0.343	0.475
Having community services	0.250	0.433	0.274	0.446	0.508	0.500	0.486	0.500
Area	1.649 *	0.844	1.802	0.888	1.622 *	0.829	1.807	0.891
**Instrumental variables**								
Talk to families	0.872 *	0.334	0.924	0.265	0.847 *	0.360	0.850	0.357
Source of housing	0.955 *	0.207	0.955	0.208	0.909 *	0.288	0.933	0.250
**Mediator variables**								
Living resources	0.759 *	0.427	0.784	0.411	0.772 *	0.420	0.790	0.408
Availability of medical treatment	0.919 *	0.273	0.946	0.225	0.933 *	0.250	0.944	0.231
Social activities	2.949 *	0.308	2.807	0.378	2.924 *	0.354	2.708	0.426
n	3297		4526		2922		4901	

Note: * *p* < 0.001; Contrasts were adjusted using the Bonferroni correction method for multiple comparisons.

**Table 3:** Effects of an empty nest on urban and rural elders’ ADL.

	Urban Elders				Rural Elders			
	LIML Regression		2SLS Regression		LIML Regression		2SLS Regression	
	First Stage	Second Stage	First Stage	Second Stage	First Stage	Second Stage	First Stage	Second Stage
Talks to family	−0.101 ***	-	−0.101 ***	-	−0.137 ***	-	−0.137 ***	-
Source of housing	−0.030	-	−0.030	-	−0.064 **	-	−0.064 **	-
Empty nest	-	−0.617 ***	-	−0.616 ***	-	−0.238 ***	-	−0.232 ***
Gender	0.045 **	0.033 *	0.045 **	0.033 **	0.062 ***	0.023 ***	0.062 ***	0.023 ***
Age	−0.005 ***	−0.008 ***	−0.005 ***	−0.008 ***	−0.006 ***	−0.005 ***	−0.006 ***	−0.005 ***
Marital status	0.269 ***	0.159 ***	0.269 ***	0.159 ***	0.232 ***	0.049 ***	0.232 ***	0.047 ***
Education years	−0.001	−0.001	−0.001	−0.001	−0.004 *	−0.002 **	−0.004 *	−0.002 **
Main source of income	0.069 ***	0.023	0.069 ***	0.023	0.057 ***	0.014	0.057 ***	0.014
Income	−0.076 ***	−0.050 ***	−0.076 ***	−0.050 ***	−0.074 ***	−0.021 ***	−0.074 ***	−0.020 ***
Medical insurance	−0.003	−0.019 *	−0.003	−0.019 *	−0.020 *	−0.044 ***	−0.020*	−0.044 ***
Sickness in the past two weeks	−0.032 *	−0.081 ***	−0.032 *	−0.080 ***	−0.006	−0.051 ***	−0.006	−0.051 ***
Smoking	−0.041 *	−0.016	−0.041 *	−0.016	−0.019	0.009	−0.019	0.009
Drinking alcohol	−0.007	0.019	−0.007	0.019	−0.001	0.009	−0.001	0.009
Exercising	0.030 *	0.089 ***	0.030 *	0.089 ***	0.024 *	0.051 ***	0.024*	0.051 ***
Having community services	0.010	−0.003	0.010	−0.003	−0.007	−0.008	−0.007	−0.008 *
Central area	−0.025	−0.027	−0.025	−0.027 *	−0.088 ***	−0.027 ***	−0.088 ***	−0.027 ***
West area	−0.083 ***	−0.031	−0.083 ***	−0.030 *	−0.100 ***	0.001	−0.100 ***	0.002
_cons	1.544 ***	4.118 ***	1.544 ***	4.117 ***	1.710 ***	3.553 ***	1.710 ***	3.543 ***
n	2931	2931	4892	4892				
F	13.866	97.093	37.566	168.443				
*p*	0.000	0.000	0.000	0.000				

Note: Standard errors in parentheses; * *p* < 0.05, ** *p* < 0.01, *** *p* < 0.001.

**Table 4:** Effects of an empty nest on urban and rural elders’ MMSE scores.

	Urban Elders				Rural Elders			
	LIML Regression		2SLS Regression		LIML Regression		2SLS Regression	
	First Stage					Second Stage	First Stage	Second Stage
Talks to family	−0.101 ***					-	−0.137 ***	-
Source of housing	−0.030					-	−0.064 **	-
Empty nest	-					−2.438 ***	—	−2.387 ***
Gender	0.045 **	0.224 **	0.045 **	0.213 ***	0.062 ***	0.291 ***	0.062 ***	0.288 ***
Age	−0.005 ***	−0.045 ***	−0.005 ***	Second stage	First stage	Second stage	First stage	−0.045 ***
Marital status	0.269 ***	0.931 ***	0.269 ***	-	−0.101 ***	-	−0.137 ***	0.529 ***
Education years	−0.001	0.008	−0.001	-	−0.030	-	−0.064 **	−0.001
Main source of income	0.069 ***	0.260 **	0.069 ***	−3.585 ***	-	−3.333 ***	-	0.223 ***
Income	−0.076 ***	−0.278 ***	−0.076 ***	−0.259 ***	−0.074 ***	−0.195 ***	−0.074 ***	−0.191 ***
Medical insurance	−0.003	0.030	−0.003	0.030	−0.020*	−0.025	−0.020 *	−0.024
Sickness in the past two weeks	−0.032 *	−0.206 **	−0.032 *	−0.200 ***	−0.006	−0.124 ***	−0.006	−0.124 ***
Smoking	−0.041 *	−0.148	−0.041 *	−0.138 *	−0.019	−0.006	−0.019	−0.005
Drinking alcohol	−0.007	0.015	−0.007	0.017	−0.001	−0.010	−0.001	−0.010
Exercising	0.030 *	0.326 ***	0.030 *	0.318 ***	0.024 *	0.204 ***	0.024 *	0.202 ***
Having community services	0.010	0.034	0.010	0.031	−0.007	0.026	−0.007	0.026
Central area	−0.025	−0.093	−0.025	−0.086	−0.088 ***	−0.348 ***	−0.088 ***	−0.343 ***
West area	−0.083 ***	−0.247 *	−0.083 ***	−0.227 **	−0.100 ***	−0.300 ***	−0.100 ***	−0.295 ***
_cons	1.544 ***	10.005 ***	1.544 ***	9.640 ***	1.710 ***	9.063 ***	1.710 ***	8.983 ***
n	2931		2931		4892		4892	
F	12.526		97.093		42.178		168.443	
*p*	0.000		0.000		0.000		0.000	

Note: Standard errors in parentheses; * *p* < 0.05, ** *p* < 0.01, *** *p* < 0.001.

**Table 5:** Effects of an empty nest on urban and rural elders’ psychological health.

	Urban Elders				Rural Elders			
	LIML Regression		2SLS Regression		LIML Regression		2SLS Regression	
	First Stage	Second Stage	First Stage	Second Stage	First Stage	Second Stage	First Stage	Second Stage
Talks to family	−0.101 ***	-	−0.101 ***	-	−0.137 ***	-	−0.137 ***	-
Source of housing	−0.030	-	−0.030	-	−0.064 **	-	−0.064 **	-
Empty nest	-	−3.751 ***	-	−3.503 ***	-	−2.595 ***	-	−2.496 ***
Gender	0.045 **	0.185 *	0.045 **	0.173 **	0.062 ***	0.218 ***	0.062 ***	0.211 ***
Age	−0.005 ***	−0.037 ***	−0.005 ***	−0.036 ***	−0.006 ***	−0.037 ***	−0.006 ***	−0.036 ***
Marital status	0.269 ***	0.979 ***	0.269 ***	0.916 ***	0.232 ***	0.587 ***	0.232 ***	0.567 ***
Education years	−0.001	0.007	−0.001	0.007	−0.004 *	−0.005	−0.004 *	−0.005
Main source of income	0.069 ***	0.303 **	0.069 ***	0.285 ***	0.057 ***	0.219 ***	0.057 ***	0.213 ***
Income	−0.076 ***	−0.287 ***	−0.076 ***	−0.268 ***	−0.074 ***	−0.195 ***	−0.074 ***	−0.187 ***
Medical insurance	−0.003	0.034	−0.003	0.035	−0.020 *	0.011	−0.020 *	0.013
Sickness in the past two weeks	−0.032 *	−0.238 ***	−0.032 *	−0.232 ***	−0.006	−0.151 ***	−0.006	−0.152 ***
Smoking	−0.041 *	−0.157	−0.041 *	−0.147 *	−0.019	0.001	−0.019	0.003
Drinking alcohol	−0.007	0.015	−0.007	0.017	−0.001	0.018	−0.001	0.018
Exercising	0.030 *	0.309 ***	0.030 *	0.302 ***	0.024 *	0.208 ***	0.024 *	0.205 ***
Having community services	0.010	0.034	0.010	0.032	−0.007	0.010	−0.007	0.011
Central area	−0.025	−0.117	−0.025	−0.110	−0.088 ***	−0.367 ***	−0.088 ***	−0.358 ***
West area	−0.083 ***	−0.257 *	−0.083 ***	−0.237 **	−0.100 ***	−0.300 ***	−0.100 ***	−0.290 ***
_cons	1.544 ***	9.474 ***	1.544 ***	9.116 ***	1.710 ***	8.397 ***	1.710 ***	8.241 ***
n	2931		2931		4892		4892	
F	7.010		97.093		22.155		168.443	
*p*	0.000		0.000		0.000		0.000	

Note: Standard errors in parentheses; * *p* < 0.05, ** *p* < 0.01, *** *p* < 0.001.

**Table 6:** Interaction analysis between two factors.

	Rural	Urban	Female	Male	65–74 Years of Age	Over 75 Years of Age	Single	Married
Female	55.21	53.92						
Male	44.79	46.08						
*p*	0.117							
65–74 years of age	23.71	24.48	20.78	27.90				
Over 75 years of age	76.29	75.52	79.22	72.10				
*p*	0.277		0.000					
Single	60.25	56.70	74.53	40.05	25.81	69.38		
Married	39.75	43.30	25.47	59.95	74.19	30.62		
*p*	0.000		0.000		0.000			
Does not exercise	72.22	53.24	70.43	58.68	56.70	67.77	69.42	58.92
Exercises	27.78	46.76	29.57	41.32	43.30	32.23	30.58	41.08
*p*	0.000		0.000		0.000		0.000	

**Table 7:** Effect of an empty nest on elders’ health in different groups.

	Dependent Variable: ADL = 1		Dependent Variable: MMSE = 1		Dependent Variable: PH	
By gender	Male	Female	Male	Female	Male	Female
Urban	−1.011	−0.472 **	−5.791	−2.727 ***	−5.633	−3.008 ***
Rural	−0.229 *	−0.237 ***	−2.672 ***	−2.355 ***	−2.807 **	−2.538 ***
By age	65–74 years of age	Over 75 years of age	65–74 years of age	Over 75 years of age	65–74 years of age	Over 75 years of age
Urban	−0.299	−0.681 **	−1.489	−3.966 ***	−0.964	−4.315 ***
Rural	−0.038	−0.287 ***	−0.523 *	−2.906 ***	−0.426	−3.121 ***
By marriage	Married	Single	Married	Single	Married	Single
Urban	0.822	−0.448 ***	2.586	−2.623 ***	2.315 *	−2.749 ***
Rural	0.641	−0.200 ***	5.546	−2.171 ***	4.945	−2.311 ***
By exercise	Exercises	Does not exercise	Exercises	Does not exercise	Exercises	Does not exercise
Urban	−0.088	−1.031 **	−0.736	−6.175 **	−0.520	−6.664 **
Rural	0.001	−0.320 ***	−1.550 **	−2.738 ***	−1.522 **	−2.967 ***

Note: Standard errors in parentheses; * *p* < 0.1, ** *p* < 0.05, *** *p* < 0.001.

**Table 8:** Mediating effect between an empty nest and elders’ health.

Variables	Urban Area			Rural Area		
	Sobel Test (Z)	Direct Effect	Mediating Effect	Sobel Test (Z)	Direct Effect	Mediating Effect
**ADL**		−1.662 ***			−0.629 ***	
Living resources	−2.715 **		4.21%	−3.211 ***		4.74%
Availability of medical treatment	−2.527 **		6.51%	−2.082 *		4.11%
Social activities	−1.597		-	−3.371 ***		15.87%
**MMSE**		−1.474 ***			−1.172 ***	
Living resources	−2.858 **		4.36%	−4.186 ***		3.61%
Availability of medical treatment	−2.380 *		4.84%	−3.874 ***		4.12%
Social activities	−1.598		-	−3.381 ***		9.08%
**PH**		−1.976 **			−1.406 ***	
Living resources	−3.388 ***		7.68%	−5.431 ***		7.69%
Availability of medical treatment	−3.616 ***		8.33%	−5.505 ***		7.43%
Social activities	−1.596		-	−3.361 ***		5.79%

Note: (1) Standard errors in parentheses; (2) * *p* < 0.1, ** *p* < 0.05, *** *p* < 0.001; (3) Mediation effect calculation: $E_{i}=\left(\beta_{ia}*\beta_{ib}\right)/\beta_{ic}\;E_{i}$ represents the mediating effect; $\beta_{ia}$ represents the coefficient of the effect of an empty nest on mediator variables; $\beta_{ib}$ represents the coefficient of the mediator variables on elders’ health; $\beta_{ic}$ represents the direct coefficient of the effect of an empty nest on elders’ health.
